# Host cell invasion and oral infection by *Trypanosoma cruzi* strains of genetic groups TcI and TcIV from chagasic patients

**DOI:** 10.1186/s13071-016-1455-z

**Published:** 2016-04-01

**Authors:** Fernando Yukio Maeda, Tatiana Mordente Clemente, Silene Macedo, Cristian Cortez, Nobuko Yoshida

**Affiliations:** Departamento de Microbiologia, Imunologia e Parasitologia, Universidade Federal de São Paulo, São Paulo, Brazil

**Keywords:** *Trypanosoma cruzi*, TcI and TcIV lineages, Metacyclic trypomastigotes, Oral infection, Host cell invasion

## Abstract

**Background:**

Outbreaks of acute Chagas disease by oral infection have been reported frequently over the last ten years, with higher incidence in northern South America, where *Trypanosoma cruzi* lineage TcI predominates, being responsible for the major cause of resurgent human disease, and a small percentage is identified as TcIV. Mechanisms of oral infection and host-cell invasion by these parasites are poorly understood. To address that question, we analyzed *T. cruzi* strains isolated from chagasic patients in Venezuela, Guatemala and Brazil.

**Methods:**

*Trypanosoma cruzi* metacyclic trypomastigotes were orally inoculated into mice. The mouse stomach collected four days later, as well as the stomach and the heart collected 30 days post-infection, were processed for histological analysis. Assays to mimic parasite migration through the gastric mucus layer were performed by counting the parasites that traversed gastric mucin-coated transwell filters. For cell invasion assays, human epithelial HeLa cells were incubated with metacyclic forms and the number of internalized parasites was counted.

**Results:**

All TcI and TcIV *T. cruzi* strains were poorly infective by the oral route. Parasites were either undetectable or were detected in small numbers in the mouse stomach four days post oral administration. Replicating parasites were found in the stomach and/or in the heart 30 days post-infection. As compared to TcI lineage, the migration capacity of TcIV parasites through the gastric mucin-coated filter was higher but lower than that exhibited by TcVI metacyclic forms previously shown to be highly infective by the oral route. Expression of pepsin-resistant gp90, the surface molecule that downregulates cell invasion, was higher in TcI than in TcIV parasites and, accordingly, the invasion capacity of TcIV metacyclic forms was higher. Gp90 molecules spontaneously released by TcI metacyclic forms inhibited the parasite entry into host cells. TcI parasites exhibited low intracellular replication rate.

**Conclusions:**

Our findings indicate that the poor capacity of TcI lineage, and to a lesser degree of TcIV parasites, in invading gastric epithelium after oral infection of mice may be associated with the inefficiency of metacyclic forms, in particular of TcI parasites, to migrate through the gastric mucus layer, to invade target epithelial cells and to replicate intracellularly.

**Electronic supplementary material:**

The online version of this article (doi:10.1186/s13071-016-1455-z) contains supplementary material, which is available to authorized users.

## Background

The outcome of infection by *Trypanosoma cruzi*, the agent of Chagas disease, may vary greatly: 20-30 % of chronically infected patients develop severe myocarditis, alterations of the digestive tract (megaesophagus and/or megacolon) occur less frequently, whereas the majority remains asymptomatic for life. The genetic background and the immunological status of the host as well as the heterogeneity of the parasite population may contribute to this diversity [1] and possibly the route of infection and the infectious dose. According to the intraspecific nomenclature established in 2009, *T. cruzi* strains are classified into six discrete typing units (DTUs), TcI-TcVI [2]. In countries North of South America and in the Amazon region, where megaesophagus and megacolon are rare and chagasic cardiomyopathy is commonplace, TcI is the predominant agent of Chagas disease, in contrast to TcII, which has been isolated from most patients in Brazilian central east region where *T. cruzi* infection is usually associated with mega syndromes [1, 3]. TcI has been pointed out as the major cause of resurgent human disease in northern South America, based on genotyping for fine-scale resolution of the geographic distribution, using a large panel of polymorphic microsatellite markers [4]. In recent years, outbreaks of acute Chagas diseases by oral infection have been reported in Venezuela [5, 6], Colombia [7, 8] and in the Brazilian Amazon [9, 10], where TcI is prevalent and a small percentage has been identified as TcIV.

Metacyclic trypomastigotes are implicated in the above referred cases of oral *T. cruzi* infection. Based on the presence of amastigote nests within sections of gastric mucosa, but no evidence for *T. cruzi* invasion within the oropharynx or esophagus of animals challenged orally with insect-derived metacyclic forms, the invasion of gastric mucosal epithelium was suggested to be a unique portal of entry for systemic *T. cruzi* infection [11]. Experiments with TcII and TcVI strains from chagasic patients have revealed that the metacyclic stage-specific surface molecules gp82 and gp90, which act as promoter and inhibitor of target cell invasion, respectively [12, 13], play pivotal roles in oral infection [14, 15]. Gp82, which selectively binds to gastric mucin [16] is highly conserved between genetically divergent *T. cruzi* lineages [17] and is resistant to digestion by pepsin at acidic pH, whereas gp90 isoforms with differential susceptibility to peptic digestion may be expressed in different strains so that high or low infectivity by the oral route is associated with the expression of pepsin-susceptible or pepsin-resistant gp90 isoform [14, 15]. Whether such a diversity in gp90 expression and oral infectivity is also found in TcI and TcIV DTUs remains to be investigated. There is no information on experimental oral infection by these parasites, the available data refer to mice injected with blood trypomastigotes or metacyclic forms by the intraperitoneal route [18] which is an unnatural mode of infection. Here we analyzed metacyclic trypomastigotes of TcI and TcIV strains isolated from chagasic patients in different geographical regions, as regards the gp82 and gp90 expression, the ability to migrate through gastric mucin layer and to invade gastric mucosal epithelium upon oral administration into mice. To clarify the mechanisms of parasite invasion, *in vitro* cell invasion assays were performed, using human epithelial cells. As surface antigens spontaneously shed from tissue culture-derived trypomastigotes [19] have been reported to play a role in *T. cruzi* infection [20], we examined whether surface molecules were released by metacyclic forms and influenced host cell invasion.

## Methods

### Parasites and host cell invasion assay

*Trypanosoma cruzi* strains from Trypanosomatid Culture Collection (TCC), Department of Parasitology, Universidade de São Paulo, were kindly provided by Dr. Marta M.G. Teixeira. They were isolated from chagasic patients in different geographical regions: TCC:28 (Amazon, Brasil), TCC:515 (Venezuela), TCC:588 (Guatemala), TCC:1522 (Paraíba, Brasil), TCC:1434 (Amapá, Brasil). Strains 28, 515, 588 and 1522 were TcI and strain 1434, isolated from an individual infected by the oral route, was TcIV. As a control, CL strain (TcVI) was used in several experiments. Parasites were maintained cyclically in mice and in liver infusion tryptose medium. To stimulate differentiation, parasites were grown for one passage in TC100 (Vitrocell, Brazil) or Grace’s medium (Invitrogen) and metacyclic forms were purified by passage through DEAE-cellulose column, as described [21]. HeLa cells, the human carcinoma-derived epithelial cells, were grown at 37 °C in Dulbecco’s Minimum Essential Medium (DMEM), supplemented with 10 % fetal calf serum (FCS), streptomycin (100 μg/ml) and penicillin (100 U/ml) in a humidified 5 % CO_2_ atmosphere. Cell invasion assays were performed as detailed elsewhere [22], by seeding purified metacyclic trypomastigotes onto each well of 24-well plates containing 13-mm diameter round glass coverslips coated with 1.4 × 10^5^ HeLa cells, either in DMEM with 10 % FCS (D10) or in PBS^++^ (PBS containing per liter: 140 mg CaCl_2_, 400 mg KCl, 100 mg MgCl_2_.6H_2_O, 100 mg MgSO_4_.7H_2_O, 350 mg NaHCO_3_). After 1 h incubation with parasites, the duplicate coverslips were fixed in Bouin solution, stained with Giemsa, and sequentially dehydrated in acetone, a graded series of acetone:xylol (9:1, 7:3, 3:7) and xylol. The number of intracellular parasites was counted in a total of 250 cells.

### Oral infection

Five to six week-old female BALB/c mice, bred in the animal facility at Universidade Federal de São Paulo, were used. All procedures and experiments conformed with the regulation of the institutional Ethical Committee for animal experimentation, and the study was approved by the Committee (Protocol No 0234/12). Mice were infected with *T. cruzi* metacyclic forms by the oral route (5 × 10^7^ parasites in 0.1 ml PBS per animal), using 1 ml syringe with gavage needle that was inserted into the animal mouth. For detection of parasites in the gastric mucosal epithelium, the stomach of mice inoculated orally with metacyclic forms was collected 4 days post-infection, fixed with 10 % neutral formaldehyde for 24 h. After processing by gradual dehydration in a graded series of ethanol solution, followed by xylene immersion and embedding in parafin, serial 5 μm tissue sections were cut and stained with hematoxylin and eosin. In another set of experiments, metacyclic forms were given orally into mice (1 × 10^6^ parasites in 0.1 ml PBS per animal) and starting on day 10 post-inoculation, parasitemia was monitored twice a week by examining 5 μl blood samples collected from the tail, at the phase contrast microscope. At 30 day post-infection, the stomach, heart and liver were collected and processed for histological preparations, as described above.

### Assay of parasite migration through gastric mucin layer

Polycarbonate transwell filters (3 μm pores, 6.5 mm diameter, Costar) were coated with 50 μl of a preparation containing 10 mg/ml gastric mucin in water. Metacyclic forms, suspended in 600 μl PBS, were added to the bottom of 24-well plates (1 × 10^7^ parasites/well), the mucin-coated transwell filters were placed onto parasite-containing wells and 100 μl PBS were added to the filter chamber. After 30 min and/or 1 h incubation at 37 °C, 10 μl samples were collected from the filter chamber for parasite counting.

### Flow cytometry and indirect immunofluorescence assays

Live metacyclic trypomastigotes (1 × 10^7^) were incubated on ice for 1 h with monoclonal antibody 3 F6 or 1G7, directed respectively to metacyclic stage-specific surface molecule gp82 or gp90. Thereafter, the parasites were fixed with 4 % para-formaldehyde for 20 min. Following washings in PBS, the parasites were incubated with Alexa Fluor 488-conjugated anti-IgG for 1 h at room temperature and the number of fluorescent parasites was estimated with a BD AccuriTM C6 Flow Cytometer. Control parasites were incubated with the secondary antibody only. To visualize HeLa cell lysosomes, coverslips with adherent cells were incubated for 1 h at 37 °C in D10 or in PBS^++^, or were incubated with parasites in D10 for 1 h. After fixation with 4 % p-formaldehyde in PBS for 30 min, the cells were treated with 50 mM NH_4_Cl in PBS for 30 min and washed in PBS. The cells were then incubated for 1 h at room temperature with mouse anti-human LAMP2 diluted 1:8 (v/v) in a PBS solution containing 0.15 % gelatin, 0.1 % sodium azide and 1 % saponin (PGN-Saponin). After washings in PBS, the coverslips were incubated for 1 h with Alexa Fluor 568-conjugated anti-mouse IgG (Invitrogen), diluted 1:300 in PGN-Saponin containing 500 ng/ml phalloidin-FITC and 10 μg/m DAPI (4′,6′-1-diamino-2-phenylindole dihydrochloride), followed by washes in PBS and subsequent mounting of coverslips in ProLong Gold (Invitrogen). Confocal images were acquired in a Leica TCS SP8 laser-scanning microscope (Leica, Germany) using an oil immersion Plan-Apochromat 63X objective (numerical aperture 1.4). The series of images obtained from confocal z-stacks were processed and analyzed using Leica LAS AF (Leica, 2012, Germany) and Imaris (Bitplane) software.

### Preparation of parasite conditioned medium

Metacyclic forms (10^8^) were incubated at 37 °C for 1 h in 100 μl complete D10 medium, nutrient-deprived PBS^++^ or PBS. After centrifugation, the pellet was discarded and the supernatant (conditioned medium) was collected. For use in cell invasion assays the conditioned medium was diluted 1:100 in D10 or PBS^++^ and for Western blot 10 μl was loaded.

### Production and purification of recombinant gp82 protein

The recombinant protein containing the full-length *T. cruzi* gp82 sequence (GenBank^TM^ data base, accession number L14824) in frame with gluthatione S-transferase (GST), was produced in *E. coli* DH5-α and purified as detailed elsewhere [23].

### Statistical analysis

The Student’s *t* test, as implemented in GraphPad software (Version 6.01), was employed.

## Results

### Oral infection of mice with *T. cruzi* metacyclic forms

The ability of TcI parasites to infect mice by the oral route was examined in a set of experiments. Forty mice were separated in 8 groups of five animals. In one set of experiments, aimed at checking the invasion of gastric epithelium by metacyclic trypomastigotes, 4 groups were used and each group of five mice received a different parasite strain (5 × 10^7^ parasites per animal). Four days after oral administration, the stomach of mice was collected and processed for histological preparations. A high number of parasites was used in this case because in previous studies with TcI strain G, from the wild transmission cycle, we had found that even with a high inoculum very few amastigote nests, which correspond to intracellulary replicating amastigotes, were detectable at 4 day post oral infection. Despite the large inoculum, we could not detect amastigote nests by microscopic analysis of histological sections of the stomach (Table 1). Another set of experiments was performed to determine whether there was any difference in tissue tropism between *T. cruzi* strains. For that purpose, the remaining 4 groups were used and each group of five mice received a different parasite strain (1 × 10^6^ parasites per animal) and the course of infection was followed for 30 days. Starting on day 10, the parasitemia levels were monitored twice a week, and at day 30 the stomach, heart and liver were collected for histological analysis. In this case, the inoculum was smaller because we reasoned that, after several rounds of cell invasion and intracellular replication, amastigote nests would be detectable. Parasitemia was undetectable throughout the period of observation but infection was confirmed in all groups by detection of amastigote nests in the stomach and/or in the heart of mice (Table 1), but not in the liver. Of note was that in the stomach of mice infected with strain 1522, which derived from a chronic chagasic patient with end-stage heart failure [24], parasites were found in the heart but not in the stomach whereas the opposite applied to infection by strain 28 (Table 1). Amastigote nests were present at high numbers in the stomach of some mice infected with strain 28 or 588 (Table 1), presumably resulting from multiple rounds of invasion and intracellular replication in the gastric epithelium. The fact that parasites were not detected in the mouse stomach at 4 day after oral administration, but only at a later time point, after intracellular replication cycles, suggested either an inefficient migration of metacyclic forms through the mucus layer, inefficient invasion of target epithelial cells and/or intracellular parasite multiplication. In addition to TcI strains, we examined one TcIV strain, 1434, derived from an orally infected patient. Amastigote nests, in small numbers, were detected in 3 mice at 4th day post-infection in the histological sections of the stomach, but parasites were not found in the stomach or in the heart 30 days after infection (Table 1). Parasitemia was negative throughout the course of 30 day infection. Positive hemoculture confirmed infection by all strains. Histological preparations were also examined for the presence of inflammatory processes. Regardless of the parasite strain, inflammatory foci were barely detectable in the stomach at day 4 or 30 post-infection, or in the heart at day 30.

**Table 1:** Oral infection of mice with metacyclic forms of *T. cruzi* strains^a^

*T. cruzi* strain (origin)	Mouse	Amastigote nests (number of tissue sections)		
		Stomach (Day 4)	Stomach (Day 30)	Heart (Day 30)
28 (Brazil Amazon)	1	0 (20)	0 (20)	0 (20)
	2	0 (20)	60 (20)	0 (21)
	3	0 (20)	285 (20)	0 (20)
	4	0 (20)	11 (18)	0 (23)
	5	0 (20)	ND^b^	0 (20)
515 (Venezuela)	1	0 (21)	0 (24)	0 (20)
	2	0 (19)	0 (13)	0 (25)
	3	0 (20)	0 (21)	0 (23)
	4	0 (20)	45 (11)	0 (21)
	5	0 (20)	5 (21)	0 (24)
588 (Guatemala)	1	0 (09)	31 (19)	10 (21)
	2	0 (12)	17 (18)	0 (20)
	3	0 (09)	6 (20)	3 (15)
	4	0 (12)	87 (19)	0 (19)
	5	0 (16)	990 (20)	0 (16)
1522 (Brazil Northeast)	1	0 (16)	0 (17)	0 (20)
	2	0 (19)	0 (15)	4 (20)
	3	0 (20)	0 (17)	0 (20)
	4	0 (21)	0 (20)	6 (20)
	5	0 (21)	0 (16)	4 (18)
1434 (Brazil Amazon)	1	5 (40)	0 (18)	0 (20)
	2	3 (40)	0 (24)	0 (20)
	3	0 (36)	0 (20)	0 (20)
	4	0 (35)	0 (20)	0 (21)
	5	5 (40)	0 (20)	0 (21)

^a^Metacyclic forms of the indicated parasite strains were given orally into mice. In one experiment, mice received 5 × 10^7^ parasites and 4 days later the stomach was collected for histological preparations. In another experiment, mice received 1 × 10^6^ parasites and 30 days later, the stomach and the heart were collected for histological preparations

^b^ *ND* not determined

### Migration of *T. cruzi* metacyclic forms through the gastric mucin layer

**Fig. 1:**
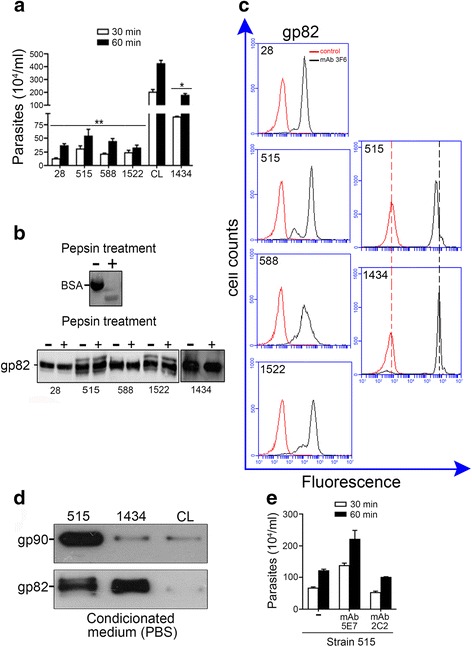
Migration of gp82-expressing *T. cruzi* metacyclic forms through the gastric mucin layer. **a** Transwell filters coated with gastric mucin were placed onto wells containing the indicated parasite strains. Samples were collected from the filter chamber at 30 min and 60 min for parasite counting. Values are the means ± SD of three independent experiments. As compared to CL strain, a significant lower migration of TcI strains (**P < 0.0005) and TcIV strain 1434 (*P < 0.005) was observed at 60 min. **b** Metacyclic forms, untreated (−) or treated (+) with 2 mg/ml pepsin, at pH 3.5, were analyzed by Western blot using mAb 3 F6 directed to gp82. As control of pepsin activity, BSA stained by Coomassie blue is shown. **c** Live parasites were incubated on ice for 1 h, in absence or in the presence of mAb 3 F6. After fixation, the parasites were incubated with Alexa Fluor 488-conjugated anti-IgG and the number of fluorescent parasites was estimated. **d** Parasites were incubated for 1 h in PBS. After centrifugation to remove parasites, the conditioned PBS was analyzed by Western blotting using mAb 3 F6 and mAb 5E7, directed to gp82 and gp90 respectively. **e** Transwell filters coated with gastric mucin were placed onto wells containing metacyclic forms of strain 515 alone, or in the presence of anti-gp90 mAb 5E7 that does not recognize live parasites or unrelated mAb 2C2. After 30 and 60 min incubation, samples from the filter chamber were taken for parasite counting. Values are the means ± variation of duplicates

An assay to mimic the parasite translocation through the mucus layer in the stomach was performed using transwell filters coated with gastric mucin, which is the main macromolecular component of the mucus. Previous studies had shown that TcVI metacyclic forms, which efficiently invade gastric epithelium when administered orally into mice, traverse the gastric mucin-coated transwell filter as effectively as the empty filter and this property is associated with the expression of pepsin-resistant g82 molecule that selectively binds to gastric mucin [16, 25]. Gastric mucin-coated transwell filters were placed on top of wells containing metacyclic forms. After 30 and 60 min incubation at 37 °C, the number of parasites that reached the upper chamber was counted. TcI parasites displayed reduced capacity to migrate through the gastric mucin layer, as compared to strain CL (TcVI) that served as control (Fig. 1a), confirming that gastric mucin acted as a barrier for migration. Metacyclic forms of TcIV strain 1434 traversed the gastric mucin-coated filters more efficiently than TcI parasites, but still at lower rates than CL strain (Fig. 1a). We examined the expression of gp82 and its resistance to pepsin in TcI and TcIV strains. Metacyclic forms were treated for 1 h with 2 mg/ml pepsin in citrate solution at pH 3.5, a condition that extensively degraded BSA (Fig. 1b), and the detergent soluble extracts were analyzed by Western blot, along with untreated parasites, using monoclonal antibody (mAb) 3 F6 directed to gp82. Before reaction with mAb 3 F6, equal loading of the parasite samples was checked by Ponceau-S staining (Additional file 1: Figure S1A). Expression of gp82, which was preserved intact after pepsin treatment, was similar in all strains, apparently at somewhat higher levels in strain 1434 than in TcI strains (Fig. 1b). As this could explain the higher migration capacity of strain 1434, we compared the gp82 expression of this strain to that of CL strain that efficiently traversed the gastric mucin-coated filter (Fig. 1a). Gp82 expression was comparable in 1434 and CL strains (Additional file 1: Figure S1B upper panel). The presence of gp82 molecules on the parasite surface was confirmed by flow cytometry analysis (Fig. 1c). To further examine whether strain 1434 expressed higher gp82 levels than TcI strains, the Western blot analysis was repeated by using strain 515 as a representative of TcI lineage. MT samples of the two strains, prepared in the same day and electrophoresed in the same SDS-PAGE gel, showed similar profile (Additional file 1: Figure S1C). From all these data and additional FACs analysis of the two strains, showing slightly higher gp82 levels in strain 1434 (Additional file 1: Figure S1D), it is not possible to conclude that the relative efficiency of strain 1434 metacyclic forms in migrating through the gastric mucin layer is due to the differential expression of gp82. As *T. cruzi* is known to spontaneously release surface molecules [19, 20, 26], parasite-shed molecules could also be responsible for the observed effects. We checked whether gp82 was shed into medium during 1 h incubation in PBS, condition used in gastric mucin migration assay. Western blot analysis of the supernatant after parasite removal, obtained as described in the methods section, revealed that strains 515 and 1434 shed considerable amounts of gp82, in contrast to CL strain that released gp82 at barely detectable levels, whereas gp90 was shed at high levels by strain 515 and at very low levels by strains 1434 and CL (Fig. 1d). If the molecules differentially released by strains 515, 1434 and CL in fact interfere with the parasite migration through the gastric mucin coat, that would explain the high and low efficiencies of strains CL and 515, respectively, as well as the intermediate ability exhibited by strain 1434 (Fig. 1a). An experiment to demonstrate the influence of released gp90 on strain 515 migration was performed by placing gastric mucin-coated transwell filters on top of wells containing metacyclic forms alone, or mixed with anti-gp90 mAb 5E7 that does not recognize live parasites or mixed with unrelated mAb 2C2 directed to *T. cruzi* amastigote molecule [27]. After 30 and 60 min incubation, samples from the filter chamber were taken for parasite counting. Parasites mixed with anti-gp90 mAb 5E7, but not those mixed with unrelated mAb 2C2, traversed the gastric mucin coat at higher numbers than control parasites (Fig. 1e), indicating that shed gp90 interferes with parasite migration through unknown mechanism that does not involve binding, provided that gp90 does not bind to gastric mucin [28]. Interference by gp82, which could not be demonstrated because anti-gp82 mAb 3 F6 binds to live parasites, would be understandable because it binds to gastric mucin. We also examined whether gp90 and gp82 were differentially released in neutral and acidic pH. Conditioned medium obtained from metacyclic forms of strain 515 incubated in PBS, pH 7.2, or in citrate buffer, pH 3.5, was analyzed by Western blot. Release of gp82 was similar at both pH, whereas gp90 shedding was lower at acidic pH (Additional file 1: Figure S1B lower panel).

### Host cell invasion by *T. cruzi* metacyclic forms

**Fig. 2:**
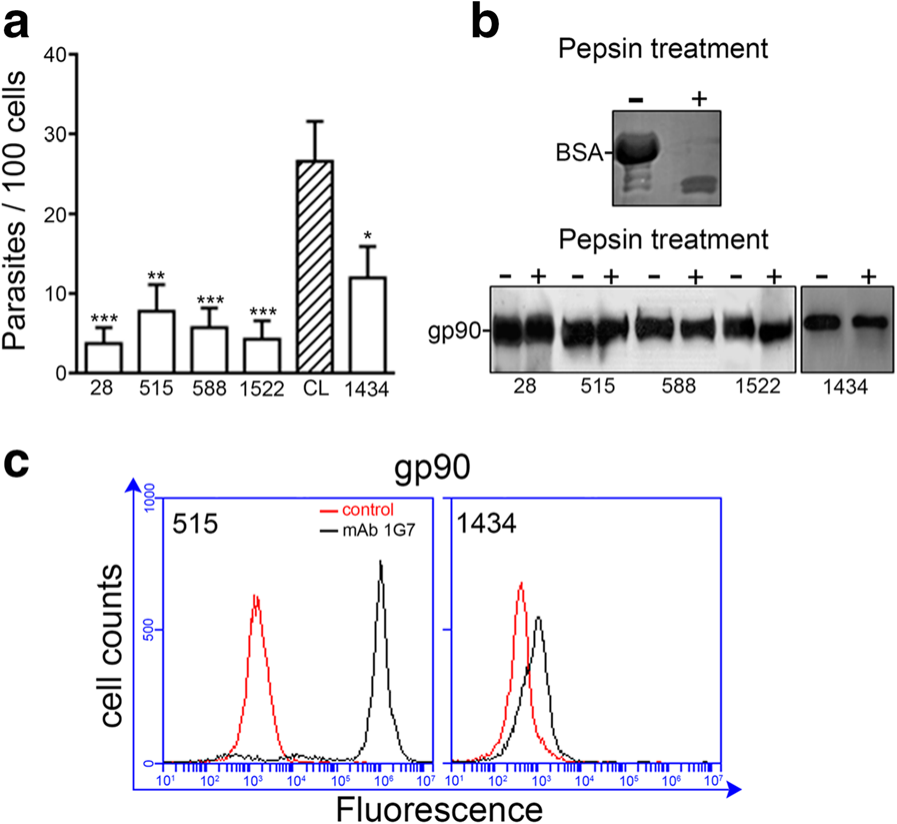
Host cell invasion by gp90-expressing *T. cruzi* metacyclic forms. **a** HeLa cells were incubated for 1 h with the indicated parasite strains in full nutrient DMEM. After fixation and Giemsa staining, the number of intracellular parasites was counted in a total of 250 cells. Values are the means ± SD of four independent assays performed in duplicate. As compared to CL strain, a significant lower invasion of strains 28, 588 and 1522 (***P < 0.0005), strain 515 (**P < 0.001) and strain 1434 (*P < 0.005) was detected by Student *t* test. **b** Metacyclic forms, untreated (−) or treated (+) with 2 mg/ml pepsin, at pH 3.5, were analyzed by Western blot using anti-gp90 mAb 1G7. As control of pepsin activity, BSA stained by Coomassie blue is shown. **c** Live parasites were incubated on ice for 1 h, in absence or in the presence of mAb 1G7. After fixation, the parasites were incubated with Alexa Fluor 488-conjugated anti-IgG and the number of fluorescent parasites was estimated

Reduced capacity of metacyclic forms in invading gastric epithelium upon oral administration into mice has been associated with expression of pepsin-resistant gp90 at high levels, and correlates with poor infectivity toward cultured human epithelial cells [14, 15]. We examined the ability of TcI and TcIV metacyclic forms to enter host cells. Parasites were incubated with HeLa cells for 1 h in full nutrient DMEM and the number of intracellular parasites was counted. Low invasion capacity was a common feature of all strains, TcIV strain 1434 strain exhibiting higher efficiency than TcI strains (Fig. 2a). Western blot analysis, using monoclonal antibody directed to gp90, revealed that all parasites expressed pepsin-resistant gp90 (Fig. 2b). The presence of gp90 on parasite surface was checked by flow cytometry analysis in strains 515 and 1434. In repeated assays using different parasite samples, gp90 was found at much lower levels in strain 1434 (Fig. 2c, Additional file 1: Figure S1E). We expected a higher cell invasion capacity of strain 1434 because the observed profile of gp90 is similar to that of CL strain metacyclic forms (Additional file 1: Figure S1B upper panel), which are not recognized by anti-gp90 monoclonal antibodies (mAbs), gp90 being detectable in detergent extract by Western blot.

### Effect of surface molecules released by *T. cruzi* metacyclic forms in host cell invasion

**Fig. 3:**
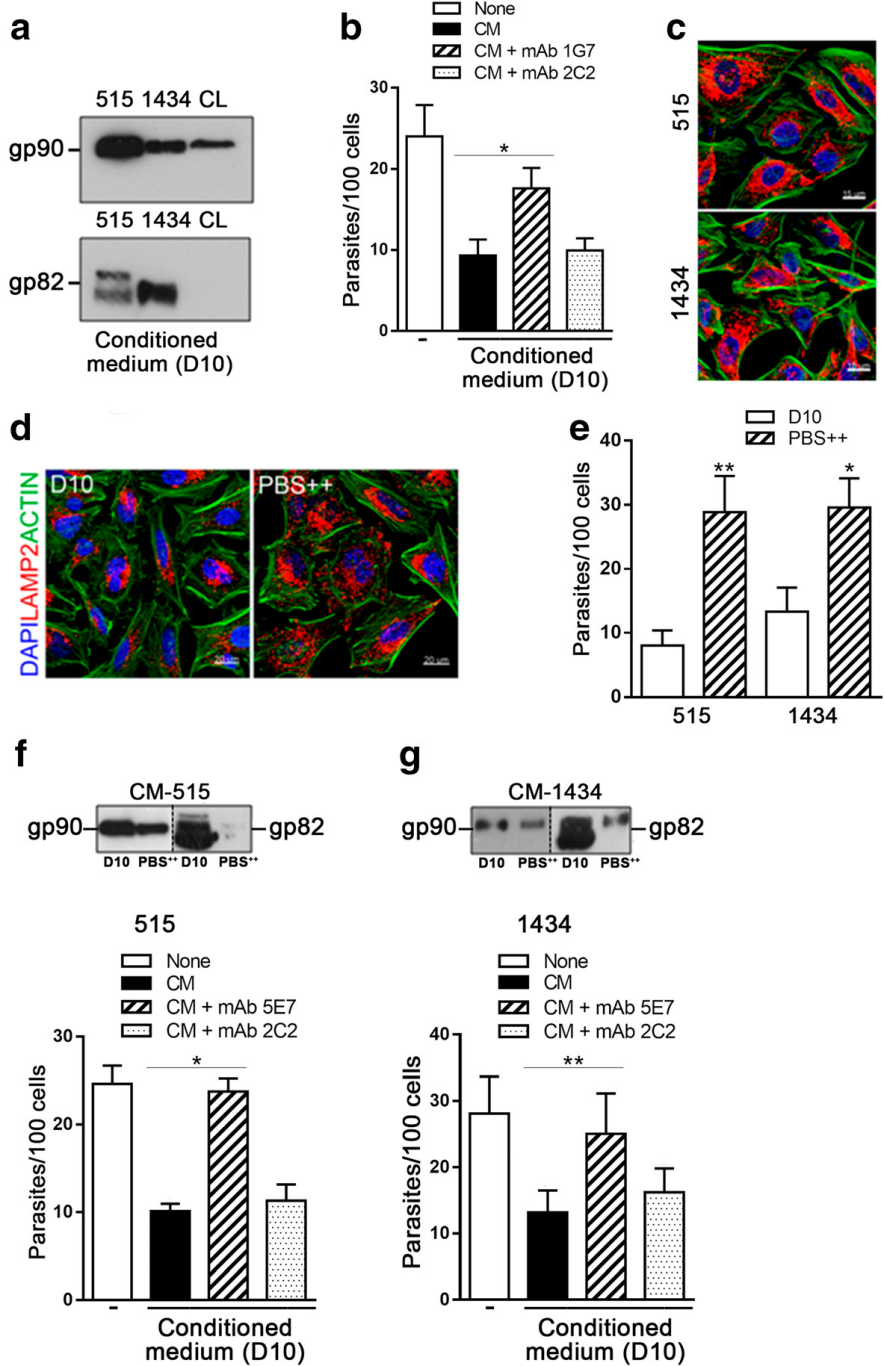
Effect of gp90 released by *T. cruzi* metacyclic forms in host cell invasion. **a** Conditioned medium of the indicated strains, prepared in D10 medium, was analyzed by Western blot. **b** HeLa cells were incubated for 1 h with CL strain metacyclic forms in D10, D10 plus conditioned medium (CM) of strain 515, prepared in D10, preincubated or not with anti-gp90 mAb 1G7 or unrelated mAb 2C2. Values are the means ± SD of three independent assays performed in duplicate. Reversal of the inhibitory effect of CM by mAb 1G7 was significant (*P < 0.05). **c** Metacyclic forms of the indicated strains were incubated for 1 with HeLa cells and then processed for confocal immunofluorescence to visualize lysosomes (*red*), actin cytoskeleton (*green*) and nucleus (*blue*). The image showed a representative microscopic field of experiment under 60X objective. Scale bar = 15 μm. **d** HeLa cells were incubated for 30 min in D10 or in PBS^++^ and processed for confocal fluorescence analysis as in (c). Note the lysosome scattering in cells incubated in PBS^++^. Scale bar = 20 μm. **e** Metacyclic forms of the strains 515 and 1434 were incubated for 1 h with HeLa cells in D10 or in PBS^++^, and then processed for Giemsa staining. Values are the means ± SD of three independent assays performed in duplicate. Cell invasion was significantly higher in PBS^++^ than in D10 for strain 515 (**P < 0.005) and strain 1434 (*P < 0.01). **f**, **g** HeLa cells were incubated for 1 h with metacyclic forms of strain 515 (**f**) or strain 1434 (**g**) in PBS^++^, PBS^++^ plus conditioned medium (CM) of respective strain prepared in D10, preincubated or not with anti-gp90 mAb 5EG7 or unrelated mAb 2C2. Values are the means ± SD of three independent assays performed in duplicate. Significant inhibition by CM was observed in invasion by strain 515 (*P < 0.05) and by strain 1434 (**P < 0.005), which could be reverted by mAb 5E7 but not by mAb 2C2. Detection of gp90 released by each strain in D10 and in PBS^++^ is shown in the upper part of the corresponding figure

We determined whether gp82 and gp90 were shed into medium during 1 h incubation in complete D10, i.e., under condition used in the cell invasion assay. Western blot analysis of the conditioned medium, prepared as described in the methods section, revealed that gp90 was shed at the highest levels by strain 515 and at the lowest levels by strain CL, whereas gp82 release by strains 515 and 1434 was clearly visualized but was barely detectable in CL strain supernatant (Fig. 3a). Next, we tested the possibility that the invasive capacity of metacyclic forms was influenced by gp90 released into the medium. CL strain metacyclic forms were incubated for 1 h with HeLa cells in D10 medium alone or in D10 plus 1 % of conditioned medium of 515 strain, preincubated or not with anti-gp90 mAb 1G7 or with unrelated mAb 2C2. CL strain was used because of its cell higher invasion capacity and lack of reaction with mAb 1G7. As shown in Fig. 3b, conditioned medium significantly reduced parasite invasion, its inhibitory activity was mostly neutralized by anti-gp90 mAb 1G7 but not by unrelated mAb 2C2. Presumably, the gp82 present in conditioned medium also contributed to the observed inhibitory effect, but this was difficult to demonstrate because anti-gp82 mAb that does not recognize live parasites is not available. In HeLa cells incubated in D10 for 30 min with metacyclic forms of strain 515 or 1434, there was some spreading of lysosomes (Fig. 3c) that may result from interaction with gp82 released to medium. The recombinant gp82 protein has been shown to induce lysosome scattering to the cell periphery [29], and event that culminates in exocytosis and contributes for parasitophorous vacuole formation required for *T. cruzi* invasion [30–32].

In another set of experiments, we examined whether molecules released by metacyclic forms of strains 515 and 1434 inhibited the host cell invasion by these parasites. As the invasion capacity of these strains is low, the assays were performed in PBS^++^, a nutrient-deprived medium that induces lysosome scattering and exocytosis [30]. Lysosomes, which are predominantly perinuclear in HeLa cells incubated in full nutrient D10, are mobilized toward cell periphery upon 30 min incubation in PBS^++^ (Fig. 3d). Invasion of metacyclic forms of strains 515 and 1434 was significantly higher in PBS^++^ than in D10 (Fig. 3e). To determine whether there was differential release of gp82 and gp90 in D10 and PBS^++^, the conditioned medium of parasites was analyzed by Western blot. Regardless of the strain, higher levels of gp82 and gp90 were released in D10 than in PBS^++^, the difference being particularly remarkable for gp82 (upper part of Fig. 3f and g). To check the effect of conditioned medium and the of gp90 on parasite invasion, HeLa cells were incubated for 1 h with metacyclic forms of strain 515 or 1434 in PBS^++^, PBS^++^ plus 1 % conditioned medium of respective strain prepared in D10, preincubated or not with anti-gp90 mAb 5EG7 or unrelated mAb 2C2. In this case we used mAb 5E7 that reacts with a cryptic epitope of gp90 and does not recognize live parasites. Significant inhibition of cell invasion of strains 515 and 1434 by respective conditioned media was observed (Figs. 3f and g). The capacity of mAb 5E7 in reverting the inhibitory effect of conditioned medium was more pronounced for strain 515 whereas unrelated mAb 2C2 had no significant effect.

### Intracellular replication of parasites

**Fig. 4:**
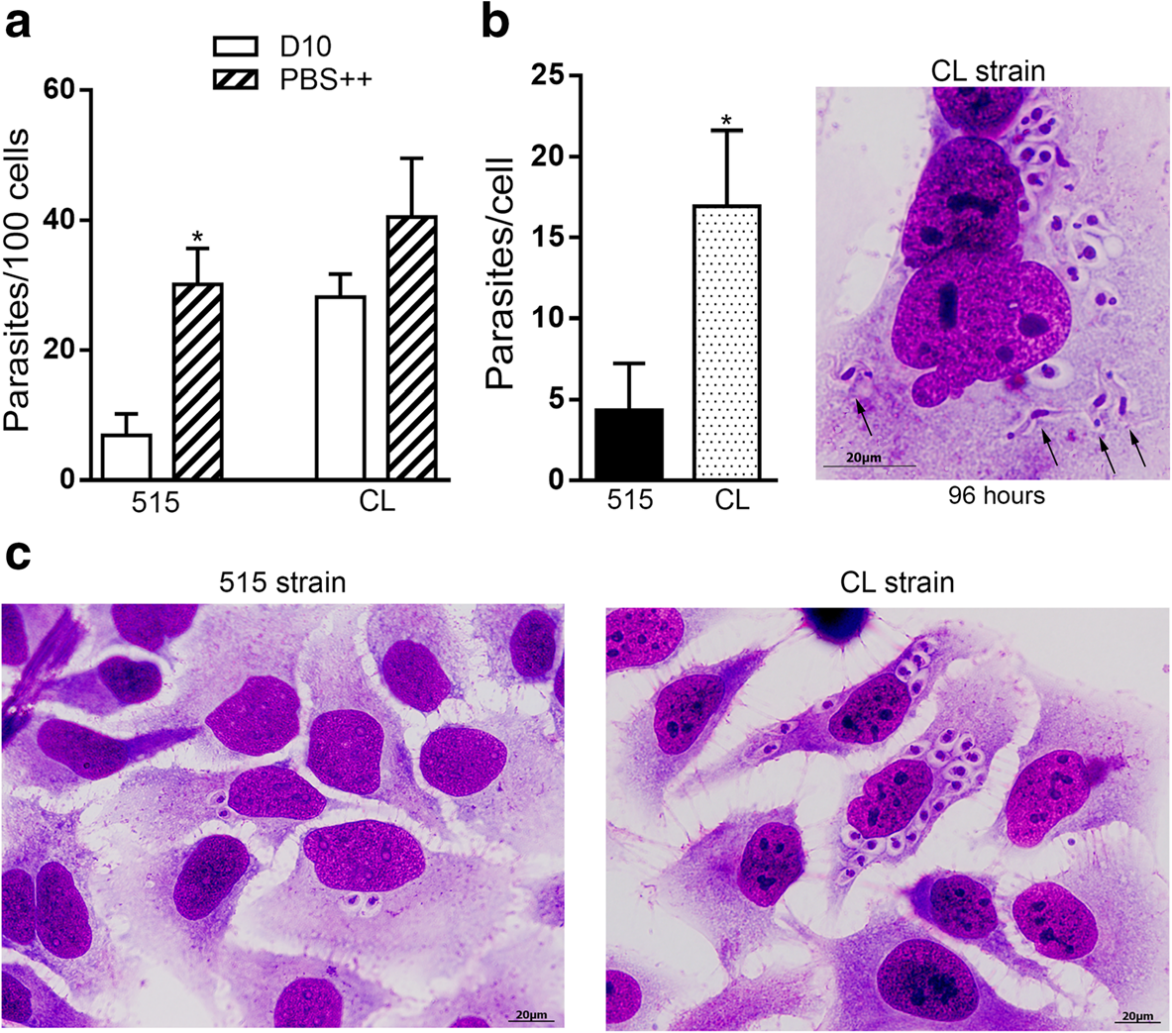
Intracellular replication of *T. cruzi*. **a** HeLa cells were incubated for 1 h with the indicated parasite strains in D10 or in PBS^++^. After fixation and Giemsa staining, the number of intracellular parasites was counted in a total of 250 cells. Values are the means ± SD of three independent assays performed in duplicate. The invasion rate of strain 515 MT was significantly higher in PBS^++^ than in D10 (*P < 0.005). **b** After 1 h incubation with MT the cells were washed to remove non internalized parasites, and maintained for 96 hs in medium with 1 % FBS. After fixation and Giemsa staining, the number of amastigotes per cell was counted. The values are the means ± SD of parasite numbers in 20 infected cells. The difference between strains 515 and CL was significant (*P < 0.005). Shown on the right is the image of a cell with amastigotes and trypomastigotes (arrows). Scale bar = 20 μm. **c** After 96 h of infection with the indicated parasite strains, HeLa cells were stained with Giemsa. Scale bar = 20 μm

In addition to cell invasion assays, experiments were performed to examine the intracellular replication of parasites, using strain 515 as representative of TcI lineage and the highly infective CL strain. Hela cells were incubated for 1 h with 515 strain MT in PBS^++^ or with CL strain MT in D10. At 1 h, the number of strain 515 MT that invaded cells in PBS^++^ was comparable to that of CL strain MT in D10 (Fig. 4a), the great majority of infected cells containing one parasite. After 1 h incubation and washings with PBS to remove non internalized parasites, the cells were maintained in DMEM containing 1 % FBS for 96 hs before processing for intracellular parasite counting. We chose 96 hs to monitor the parasite multiplication because that was the time for checking the presence of amastigote nests in the gastric epithelium after oral infection of mice. The number of intracellular parasites in cells infected with CL strain was about 4 fold higher as compared to infection by strain 515 and trypomastigotes differentiated from amastigotes could be visualized in some cells (Fig. 4b). In contrast to cells infected with CL strain, with cytoplasm full of amastigotes, most cells infected with 515 strain contained a few amastigotes (Fig. 4c).

### Involvement of gp82 and protein kinase C (PKC) in host cell invasion by *T. cruzi* metacyclic forms

**Fig. 5:**
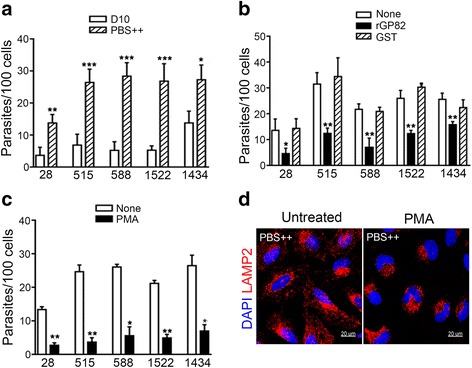
Involvement of gp82 and protein kinase C in host cell invasion by *T. cruzi* metacyclic forms. **a** HeLa cells were incubated with parasites for 1 h in full-nutrient D10 or in PBS^++^. After fixation and Giemsa staining, the number of intracellular parasites was counted in a total of 250 cells. Values are the means ± SD of three independent assays performed in duplicate. The difference in the invasion rate in D10 and PBS^++^ was significant for strains 515, 588 and 1522 (***P < 0.005), 28 (**P < 0.01), 1434 (*P < 0.05). **b** Metacyclic forms were incubated with HeLa cells in PBS^++^, in absence or in the presence of rGP82, the recombinant protein based on gp82, or GST as control. After 1 h, the cells were processed as in (**a**) for intracellular parasite counting. Values are the means ± SD of three independent assays performed in duplicate. The recombinant gp82 significantly inhibited invasion by strains 28 (*P < 0.05), 515, 588, 1522 and 1434 (**P < 0.005). **c** HeLa cells were pretreated for 30 min with PMA and then incubated with metacyclic forms in PBS^++^. After 1 h incubation, the cells were processed as in (**a**) for intracellular parasite counting. Values are the means ± SD of three independent assays performed in duplicate. Pretreatment with PMA significantly inhibited invasion of strains 28, 515, 1522 (**P < 0.0001), 588 and 1434 (*P < 0.001). **d** Hela cells, untreated or pretreated for 30 min with PMA in D10, were incubated in PBS^++^ for 30 min and processed for confocal immunofluorescence for visualization of lysosomes (*red*) and nucleus (*blue*) under 60X 0bjetive. Note the perinuclear accumulation of lysosomes in PMA-treated cells. Scale bar = 20 μm

In PBS^++^, the invasion capacity of strains 515 and 1434 reached levels comparable to that of CL strain metacyclic forms. We ascertained that other TcI strains exhibited the increased invasion capacity in PBS^++^ (Fig. 5a), suggesting that their entry into host cells is influenced by expression of surface molecules as well as by their shedding. We presumed that cell invasion by metacyclic forms of different strains in PBS^++^ is gp82-mediated. To validate the assumption, metacyclic trypomastigotes were incubated for 1 h with HeLa cells in PBS^++^, in absence or in the presence of 40 μg/ml GST or the recombinant protein containing the full-length gp82 sequence fused to GST, and the number of internalized parasite was counted. Invasion was significantly inhibited by the recombinant gp82 but not by GST (Fig. 5b). In CL strain, the gp82-mediated invasion has been associated with activation of target cell signaling pathways involving protein kinase C [30, 33]. We examined if this applied to *T. cruzi* strains analyzed here. HeLa cells in D10 were pretreated with 100 nM phorbol myristate acetate (PMA), a drug that inhibits diverse cell processes by downregulating PKC [34, 35], before incubation with parasites. As shown in Fig. 5c, pretreatment of cells with PMA significantly diminished invasion by all strains. Treatment of cells with PMA has been shown to inhibit PBS^++^-induced lysosome scattering to the cell periphery [30]. We confirmed that in cells pretreated with PMA and incubated in PBS^++^ the lysosomes remained in the perinuclear region (Fig. 5d).

## Discussion

Our previous studies had indicated that cell invasion capacity of *T. cruzi* metacyclic trypomastigotes is dependent on expression of surface molecules gp82 and gp90, which trigger opposite effects upon interaction with host cells, gp82 inducing signaling cascades leading to an increase in cytosolic Ca^2+^ concentration and lysosome scattering followed by exocytosis [29]. Metacyclic forms of *T. cruzi* strains, such as CL and Y82, which express gp82 and low gp90 levels, efficiently enter host cells *in vitro* and, when administered orally into mice, effectively invade the target gastric epithelial cells [16, 25]. The present study has revealed that gp82 and gp90 molecules that are differentially released into medium by different *T. cruzi* strains influence the cell invasion process.

We have found that during 1 h incubation in full nutrient D10 medium, condition used in our cell invasion assays, CL metacyclic forms release gp82 molecule at barely detectable levels and gp90 in small amounts, in sharp contrast with strain 515 parasites that release high amounts of gp82 and gp90. During incubation in D10 medium, gp82 and gp90 released by metacyclic forms of strain 515 presumably interfere with parasite-host cell interaction, gp82 competing with the molecule present on parasite surface for the host cell receptor, and gp90 further contributing to down modulate signaling events required for invasion. This is consistent with the wide difference in the invasion capacity of strains CL and 515. Metacyclic forms of strain 1434 expressed and released surface gp90 at levels comparable to CL strain, but their invasive capacity was much lower, which may be due to high amounts of gp82 released into medium. In contrast to CL strain, when given orally into mice, metacyclic forms of strain 515 and other TcI strains that have similar surface profile, as well as TcIV strain 1434, were poorly infective, parasites being either undetectable or detectable in small numbers in histological sections of the stomach collected 4 days post infection. The low infectivity by oral route of *T. cruzi* strains that shed gp82 and/or gp90 could be due to the inhibitory effect of these molecules on parasite invasion of gastric epithelial cells. Based on findings that the recombinant gp82 inhibits invasion by TcI/TcIV strains, we presume that gp82 released by these strains may reduce the parasite infectivity by competing for target cell binding. The premise is compatible with the finding that gp82 is released by highly invasive TcVI strains in minimal amounts, and at high levels by poorly invasive TcI strains. Why parasites of TcI/TcIV lineage release molecules that reduce their own infectivity? One idea that comes to mind is that *T. cruzi* lineage TcI/TcIV has been associated with the wild transmission cycle, and the infection of animals in nature since ancient times may have induced parasite adaptations that favored the survival of the host. Strains of *T. cruzi* lineage TcII/TcVI*,* associated with the domestic transmission cycle, have been found to be highly invasive and may kill the host [14, 25], what is disadvantageous to the parasite. Another important factor that could account for the gastric epithelium parasitism by TcI strains, which was undetectable at day 4 post oral infection, is their low intracellular multiplication rate. Cell invasion assays with TcI strain 515 showed that, at day 4 post infection of HeLa cells, a few amastigotes per cell was detected, as opposed to four-fold higher numbers in cells infected with CL strain. An additional factor that may be contributing to the observed low infective capacity is the parasite ability to traverse the mucus layer that protects the gastric mucosa. *In vitro*, the ability to migrate through gastric mucin-coat was very low in TcI strains and to a lesser degree in strain 1434. Gp82 and gp90 molecules released by parasites appear to affect translocation through gastric mucin layer. As binding of metacyclic forms is mediated by gp82, there would be a competition between surface and released gp82 molecules. The inhibitory effect of shed gp90 cannot be explained on the same basis because this molecule does not bind to gastric mucin [28].

Further support to the hypothesis that surface molecules shed into medium interfere with parasite invasion was provided by experiments with strains 515 and 1434 in nutrient-depleted PBS^++^ medium. During 1 h incubation in PBS^++^ medium, the release of gp90 and of gp82 in particular was greatly reduced, what could explain the increased cell invasion as compared to D10 medium. As a short time incubation of cells in PBS^++^ results in lysosome scattering, a condition that facilitates the parasite entry into cells, and the surface molecules are released only in small amounts, the rate of invasion reaches levels similar to those of CL strain. We found that in PBS^++^ cell invasion by all strains examined was gp82-mediated and involved the participation of protein kinase C, similarly to what was previously observed with CL strain [30].

From our findings we infer that the differential capacity of *T. cruzi* strains in invading gastric epithelium upon oral administration is associated with the differential expression and/or release of gp82 and gp90 molecules. Four TcI strains from different geographical regions were analysed and exhibited similar characteristics. As we had access to only one TcIV strain, we do not know whether the observed features apply to this DTU in general.

## Conclusions

Our findings have indicated that the poor capacity of TcI lineage, and to a lesser degree of TcIV parasites, in invading gastric epithelium after oral infection of mice may be associated with the inefficiency of metacyclic forms, in particular of TcI parasites, to migrate through the gastric mucus layer, to invade target epithelial cells and to replicate intracellularly.

## Additional file

Additional file 1: Figure S1. Expression and release of surface gp82 and g90 molecules of *T. cruzi* metacyclic trypomastigotes. **a** Ponceau-S staining of the corresponding Western blot shown in Fig. 1b, to demonstrate equal loading of metacyclic trypomastigotes samples, untreated (−) or treated (+) with 2 mg/ml pepsin, at pH 3.5. The molecular size markers are shown on the left. **b** Shown in the upper panel is the detergent extract of metacyclic forms of the indicated strains, analyzed by Western blot using monoclonal antibodies to gp82 and gp90. Shown in the lower panel is the conditioned medium obtained from strain 515 MT in PBS, pH 7.2, or in citrate buffer, pH 3.5, analyzed by Western blot. **c** Western blot of detergent extracts of strains 515 and 1434, revealed with mAb 3 F6. Shown in the lower panels is the corresponding Ponceau-S staining **d**–**e** Metacyclic forms of the indicated strains were incubated on ice for 1 h, in absence or in the presence of anti-gp82 mAb 3 F6 (**d**) or anti-gp90 mAb 1G7 (**e**). After fixation, the parasites were incubated with Alexa Fluor 488-conjugated anti-IgG and the number of fluorescent parasites was estimated. (TIF 8480 kb)
