# Supplementary figures and images for: Host cell invasion and oral infection by Trypanosoma cruzi strains of genetic groups TcI and TcIV from chagasic patients
Source: Parasit Vectors. 2016 Apr 1;9:189. doi: 10.1186/s13071-016-1455-z (PMC4818890; doi:10.1186/s13071-016-1455-z)

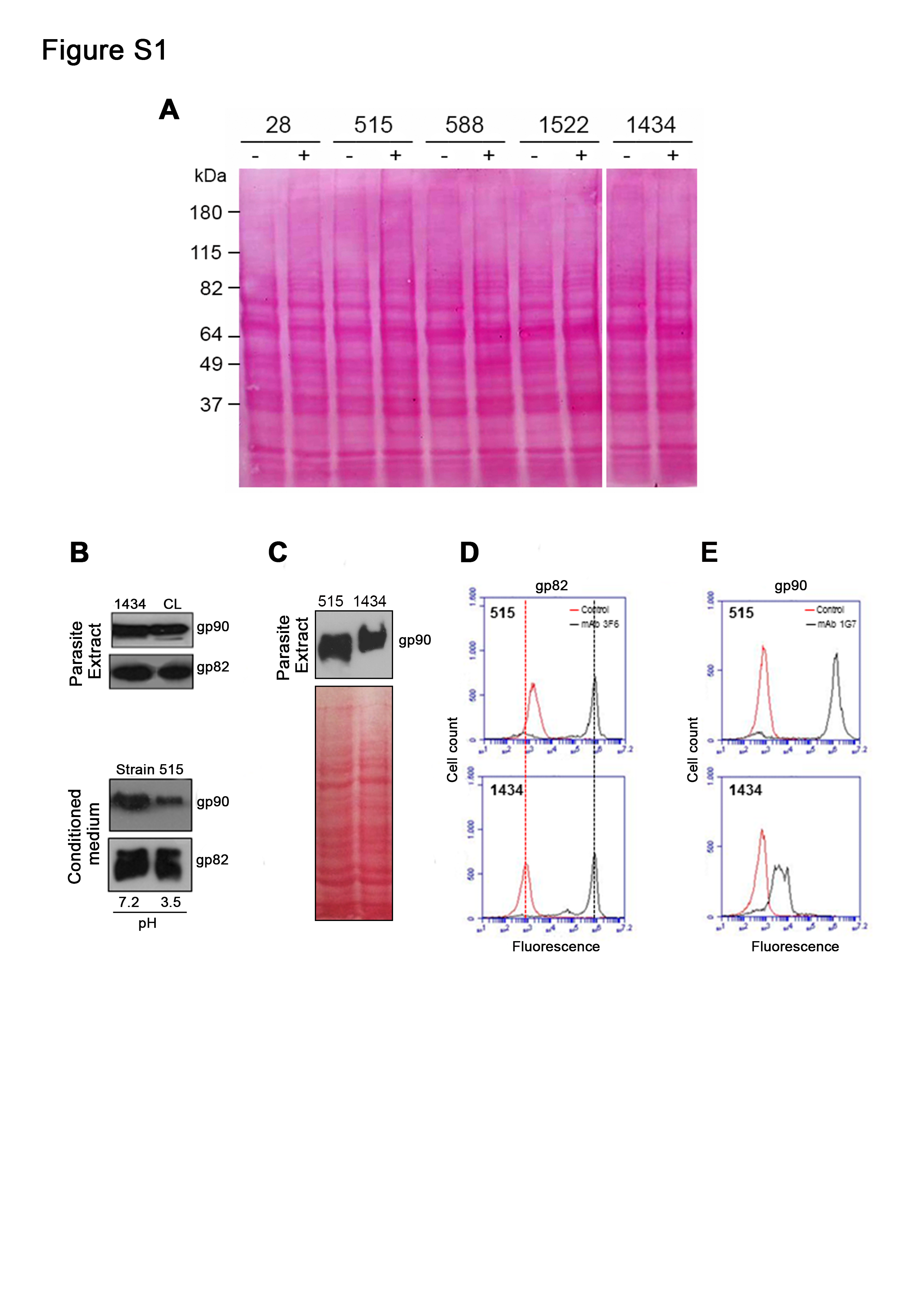

Supplement: Additional file 1: Figure S1. — Expression and release of surface gp82 and g90 molecules of T. cruzi metacyclic trypomastigotes. a Ponceau-S staining of the corresponding Western blot shown in Fig. 1b, to demonstrate equal loading of metacyclic trypomastigotes samples, untreated (−) or treated (+) with 2 mg/ml pepsin, at pH 3.5. The molecular size markers are shown on the left. b Shown in the upper panel is the detergent extract of metacyclic forms of the indicated strains, analyzed by Western blot using monoclonal antibodies to gp82 and gp90. Shown in the lower panel is the conditioned medium obtained from strain 515 MT in PBS, pH 7.2, or in citrate buffer, pH 3.5, analyzed by Western blot. c Western blot of detergent extracts of strains 515 and 1434, revealed with mAb 3 F6. Shown in the lower panels is the corresponding Ponceau-S staining d–e Metacyclic forms of the indicated strains were incubated on ice for 1 h, in absence or in the presence of anti-gp82 mAb 3 F6 (d) or anti-gp90 mAb 1G7 (e). After fixation, the parasites were incubated with Alexa Fluor 488-conjugated anti-IgG and the number of fluorescent parasites was estimated. (TIF 8480 kb) [file 13071_2016_1455_MOESM1_ESM.tif]
